# Early Warnings by Liver Organoids on Short- and Long-Chain PFAS Toxicity

**DOI:** 10.3390/toxics10020091

**Published:** 2022-02-18

**Authors:** Stefano Palazzolo, Isabella Caligiuri, Andrea Augusto Sfriso, Matteo Mauceri, Rossella Rotondo, Davide Campagnol, Vincenzo Canzonieri, Flavio Rizzolio

**Affiliations:** 1 Pathology Unit, Centro di Riferimento Oncologico di Aviano (CRO) IRCCS, 33081 Aviano, Italy; spalazzolo@cro.it (S.P.); icaligiuri@cro.it (I.C.); rossellaross1988@gmail.com (R.R.); vcanzonieri@cro.it (V.C.); 2 Department of Chemical, Pharmaceutical and Agricultural Sciences, University of Ferrara, Via Fossato di Mortara 17, 44121 Ferrara, Italy; sfrndr@unife.it; 3 Department of Molecular Sciences and Nanosystems, Ca’ Foscari University of Venice, Via Torino 155, 30170 Venice, Italy; matteo.mauceri@unive.it (M.M.); davide.campagnol@unive.it (D.C.); 4 Centro Ricerche Scientifiche, Dott. Dino Paladin, 35127 Padova, Italy; 5 Department of Medical, Surgical and Health Sciences, University of Trieste, 34127 Trieste, Italy

**Keywords:** PFAS, liver organoids, ex-vivo model, endocrine disruption

## Abstract

Short-chain per-fluoroalkyl substances (PFAS) have replaced long-chains in many applications, however the toxicity and its mode of action and interactions due to the large number of these compounds and their mixtures is still poorly understood. The paper aims to compare the effects on mouse liver organoids (target organ for bioaccumulation) of two long-chain PFAS (perfluorooctane sulfonate -PFOS-, perfluorooctanoic acid -PFOA) and two short-chain PFAS commonly utilized in the industry (heptafluorobutyric acid -HFBA-, Pentafluoropropionic anhydride-PFPA) to identify the mode of action of these classes of contaminants. Cytomorphological aberrations and ALT/GDH enzyme disruption were identified but no acute toxicity endpoint neither apoptosis was detected by the two tested short-chain PFAS. After cytomorphological analysis, it is evident that short-chain PFAS affected organoid morphology inducing a reduction of cytostructural complexity and aberrant cytological features. Conversely, EC50 values of 670 ± 30 µM and 895 ± 7 µM were measured for PFOS and PFOA, respectively, together with strong ALT/GDH enzyme disruption, caspase 3 and 7 apoptosis activation and deep loss of architectural complexity of organoids in the range of 500–1000 µM. Eventually, biochemical markers and histology analysis confirmed the sensitivity of organoid tests that could be used as a fast and reproducible platform to test many PFAS and mixtures saving time and at low cost in comparison with in vivo tests. Organoids testing could be introduced as an innovative platform to assess the toxicity to fast recognize potentially dangerous pollutants.

## 1. Introduction

Per-fluoroalkyl substances (PFAS) have been largely produced and commercialized in the second half of the 20th century [1] and have been found in many samples from urban areas to remote regions of the planet such as the Arctic trophic food webs [2,3]. This class of contaminants was recorded as ubiquitary in many environmental spheres: soil [4], groundwaters [5], surface and drinking waters [6,7,8], deep ocean [9], remote lakes, air [10] and eventually humans [11]. Long-chain PFAS such as perfluorooctane sulfonate (PFOS), perfluorooctanoic acid (PFOA) and their respective salts were listed as persistent organic pollutants under the Stockholm Convention in 2009 [12] and management strategies, regulations and risks assessments were subsequently developed in many countries [13,14,15,16]. PFOA and PFOS are two synthetic compounds employed from 1950s in various industrial products because of their chemical properties, in particular high hydrophobicity and lipophobicity. PFOA and PFOS were optimal for the production of industrial surfactants used in the manufacturing of a variety of consumer products including paints, synthetic lubricants, adhesives, stain repellents, insecticides, extinguishing foam and waterproof coating of technical tissues-e.g., goretex, nomex [17]. Short-chain PFAS are currently extensively used as an alternative to long-chain ones and, after a first period of free production and use, the European community inserted some long-chain PFAS into regulations concerning use, production and water concentration [15]. Long- and short-chains PFAS are very persistent in the environment [11], resistant to microbial degradation [18] and subjected to bioaccumulation and biomagnification in the food chain [19]. Waste-water treatment plants and carbon filtration technologies often proved to be inefficient in their removal [20] and short-chain PFAS [21] upon release in freshwater environments, the main transit ecosphere for many pollutants, displayed lower absorption in suspended solids in turn remaining longer time in the water column [22]. As a consequence, short-chain PFAS display higher mobility in the environment in comparison with their long-chain counterparts. Additionally, both long- and short-chain PFAS accumulate in the edible parts of plants and fruits [23,24]. Once in the human body, these compounds bind to plasma proteins, thus increasing their half-life in the body [25]. Since the irreversible trend of accumulation of many of these compounds, it is possible that systemic effects will be visible once a threshold value is reached [26]. The studies mentioned illustrate and highlight the risks and concerns that characterize short-chain PFAS suggesting the need of risk assessment and usage regulation [26].

In this paper, we aimed to evaluate and compare the toxicity of two long and two short-chain PFASs toxicity in a physiological relevant and innovative liver mini-organs (organoids) at cellular level by measuring cell viability, structural histology and enzymatic activity. Organoids are 3D models that can recapitulate in vitro the physiology of organs and represent a platform in which it is possible to predict the effects of a compound more rapidly than in vivo [27,28,29,30,31]. Two long-chain (PFOS, PFOA) and two short-chain PFAS (HFBA: Heptafluorobutyric acid also known as perfluorobutyric acid; PFPA: Pentafluoropropionic anhydride) were tested.

## 2. Materials and Methods

### 2.1. Reagents

PFOS (purity ≥ 98.0% CAS: 2795-39-3, #77282, Merck Sigma-Aldrich, Darmstadt, Germany), PFOA (purity 95%, CAS: 335-67-1, #171468, Merck Sigma-Aldrich, Darmstadt, Germany), PFPA (purity 97%, CAS: 356-42-3, #77292, Merck Sigma-Aldrich, Darmstadt, Germany), HFBA (purity 98%, CAS: 375-22-4, #164194, Merck Sigma-Aldrich, Darmstadt, Germany).

### 2.2. Organoid Isolation

Mouse liver organoids were isolated by C57/BL6 mouse 6 weeks old following the protocol described by Miyoshi and Stappenbeck [32] adapted by Palazzolo et al. [33]. Briefly, organoids were obtained after the enzymatic digestion of a fragment of mouse liver by collagenase I (Thermo Fisher, Waltham, MA, USA). The group of cells obtained were seeded in a 3D matrtix (coultrex Type 3, Bio-Techne, Minneapolis, MN, USA) in a drop of 10 µL in a 24 multiwell plate. Organoids were generated from mouse liver post mortem (Italian Ministry of Health, 148/2016-PR, accepted on 19 February 2016 and renewed on 25 March 2019).

### 2.3. Cell Viability Assay (EC50, Effective Concentration 50)

Mouse liver organoids were seeded in 96-wells plates (Becton Dickinson, Franklin Lakes, NJ, USA) and incubated for 48 h to allow the formation of organoids and treated with the same drug concentrations for 96 h. Mouse liver organoids were treated with 2 mM, 1 mM and serial dilution (1:10) to 0.1 μM of selected PFAS. PFAS stock were dissolved in DMSO to a final concentration of 100 mM, serial dilution for the treatment in every experiment were done in organoids culture medium. The cytotoxicity was evaluated by CellTiter-Glo^®^ Luminescence assay (Promega, WI, USA) with an Infinite200 PRO instrument (Tecan, Switzerland) after 96 h. EC50 values were calculated with Graph Pad Prism 4.0 (Graph Pad Software Corporation, San Diego, CA, USA) with a sigmoidal dose-response curve.

### 2.4. Histological and Immunohistochemical Staining

According to previously reported cytomorphological evidences, microscopic examination of H&E-staining revealed that liver organoids show the typical features of hepatic progenitors and mature hepatocytes [34]. Organoids were washed with PBS and embedded using Bio-Agar (Bio-Optica, Milano, Italy). Subsequently, the blocks were fixed in 10% neutral buffered formalin overnight and processed for paraffin embedding. Sections of 2.5 µm thickness were used for hematoxylin and eosin (H&E) staining by using Leica ST5020 Multistainer. Mouse liver organoids were evaluated and analyzed untreated and treated with 100, 500, 1000 µM selected PFAS and the induction of morphological changes evaluated at 6, 24 and 48 h by histological analysis.

### 2.5. ALT and GDH Activity Assay

Alanine aminotransferase (ALT) and glutamate dehydrogenase (GDH) levels increase in the blood after liver injury [35] and were chose as bioindicators to asses liver enzymatic functionality. Mouse liver organoids were seeded in 24-wells plates (Becton Dickinson, NJ, USA) and incubated for 48 h to allow the formation of organoids and treated with PFASs 100, 500, 1000 µM. Medium was collected after 1, 3 and 6 h and analyzed with Alanine Aminotransferase (ALT) Activity Assay and Glutamate Dehydrogenase (GDH) Activity Assay Kit (Merck Sigma-Aldrich, Darmstadt, Germany) following the respective supplier protocol. Fluorescence (535/587 nm for ALT assay) and absorbance (480 nm for GDH assay) were measured with an Infinite 200 PRO instrument (Tecan, Menendorf, Switzerland).

### 2.6. Caspases 3/7 Activity

Mouse liver organoids were seeded in 96-wells plates (Becton Dickinson, NJ, USA) and incubated for 48 h to allow the formation of organoids and treated with PFASs 100, 500, 1000 µM. The caspases 3/7 activity was evaluated with Caspase-Glo^®^ 3/7 Assay System (Promega, Madison, WI, USA) with an Infinite 200 PRO instrument (Tecan, Switzerland) after 1 and 3 h.

### 2.7. Statistical Analysis

The statistical significance was determined using a two-tailed *t*-test. A *p* value less than 0.05 was considered significant for all comparisons done. Bars represent standard deviations.

## 3. Results

### 3.1. Effects of PFAS on Mouse Liver Organoids Viability

Cell viability was tested starting from a concentration of 1 mM. In general, PFOS and PFOA were more toxic than short chain PFAS, as both HFBA and PFPA displayed no cell viability inhibition in the tested concentration range (100–1000 µM). PFOS and PFOA showed EC50 values of 670 ± 30 µM and 895 ± 7 µM, respectively (Table 1).

### 3.2. Structural Alterations in Liver Organoids

**Figure 1:**
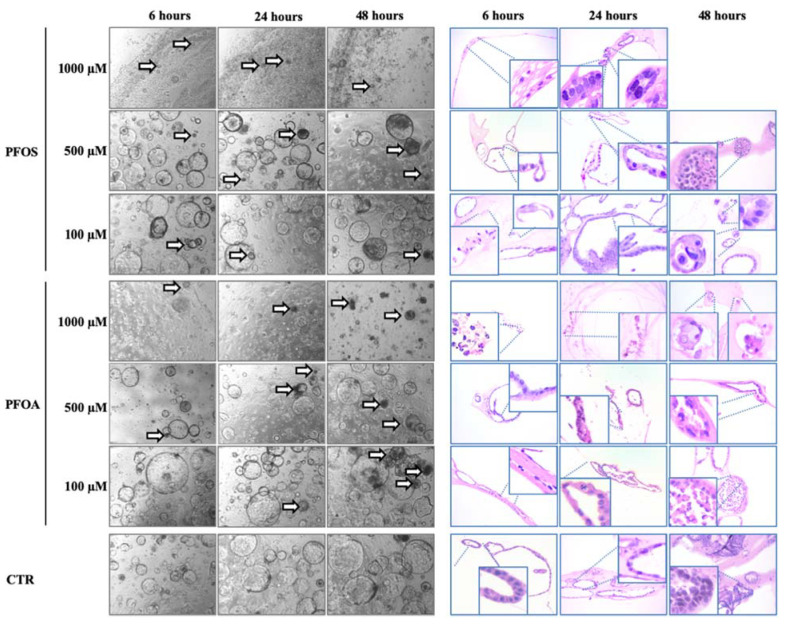
Morphological changes of mouse liver organoids treated with different concentrations of PFAS. Mouse liver organoids treated with PFOS and PFOA for 6, 24 and 48 h at 1 mM, 500 µM and 100 µM. Images show morphological changes of organoids in bright field and H/E staining. Arrows indicate dead or suffering organoids. Empty panels are due to the toxicity of the treatment that induced organoids death. Zoom areas highlight organoids cellular and nuclear damages. H/E magnification 20×; bright field magnification 4×.

Morphological examination of H&E-staining revealed that the treatments of mouse liver organoids with long chain PFOS and PFOA compounds induced cytostructural changes (Figure 1). In particular, compared with controls, the highest concentration of PFOS and PFOA (1000 μM) induced an early loss of epithelial cells and of architectural complexity of organoids, visible just after 6 h of exposure to these compounds. After 24 h of treatment, PFOS and PFOA at a concentration of 1000 μM induced nuclear pyknosis and a complete loss of cell architecture. The effect became more marked after 48 h of exposure, organoids were severely and remarkable degenerated, with undetectable presence of organoids or residual single cells. Conversely, untreated mouse liver organoids appeared as well-organized structures without cellular atypia, that proliferated to form stratified epithelial structures. Compared to the controls, treatment of organoids with PFOS and PFOA at 500 µM induced an overall reduction of cytostructural complexity with the appearance of nuclear pyknosis after 24 and 48 h of treatment. Morphological changes resulted less pronounced when organoids were treated with a 10 times lower concentration (100 µM), since their structural complexity was partially conserved. However, karyorrhectic nuclear debris and cellular disintegration were still visible after 6 h of exposure to PFOS and PFOA respectively as well as cellular alteration, loss of polarity and nuclear pyknosis at long-term exposure of 24 and 48 h.

**Figure 2:**
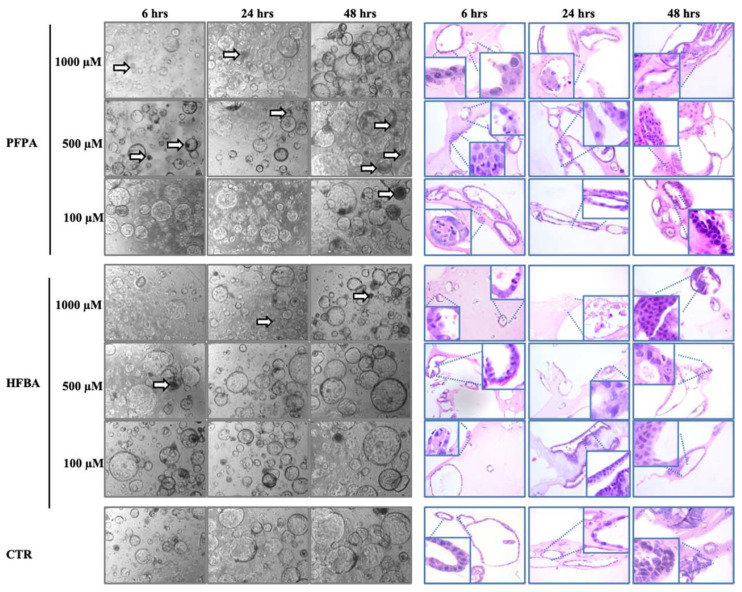
Morphological changes of mouse liver organoids treated with different concentrations of PFAS. Mouse liver organoids treated with HFBA and PFPA for 6, 24 and 48 h with 1 mM, 500 µM and 100 µM. Images show morphological changes of organoids in bright field and H/E staining. Arrows indicate dead or suffering organoids. Zoom areas highlight organoids cellular and nuclear damages. H/E magnification 20×; bright field magnification 4×.

Similar to the EC50 experiments, the analysis with light microscopy revealed a similar growth for organoids treated with PFPA and HFBA with respect to untreated organoids although minor significant cytotoxic effects were observed (Figure 2). The effects of HFBA seem to be more pronounced if compared with PFPA, since a general reduction of cytostructural complexity has been observed in organoids treated with HFBA at 1000 μM at different time points (6, 24, 48 h). In detail, the initial nuclear hyperchromasia and reduced structural organization observed at 6 h of treatment were followed by loss of cells and architectural organization at 24 h and by reduction of cytostructural complexity after 48 h of HFBA exposure.

Cytomorphological changes were appreciable in mouse liver organoids when treated at 500 μM and 100 μM. In particular, the structural complexity was still partially conserved at 6 h of treatment at 500 μM, but a reduction of cytostructural complexity and nuclear pyknosis became evident at 24 h of exposure as well as cytolysis and pyknosis at 48 h of treatment. Except for the presence of karyorrhexis at 6 h of treatment, conserved complexity and preserved structural organization were still visible when organoids were treated with HFBA at 100 μM for 24 and 48 h. Despite the structural organization were partially conserved after 48 h of treatment with PFPA at 1000 μM, some aberrant cytological features such as nuclear hyperchromasia and cytolysis have been evidenced after 6 and 24 h of treatment. At lower concentrations of PFPA (500 μM and 100 μM), the structural complexity of treated organoids resulted partially conserved at 6, 24 and 48 h with the appearance of hyperchromasia at 6 h of treatment with PFPA at 500 μM and scattered hyperchromatic nuclei at 6 and 48 h of exposure with PFPA at 100 μM.

### 3.3. Long and Short PFASs Alter the Activity of Key Liver Enzymes

**Figure 3:**
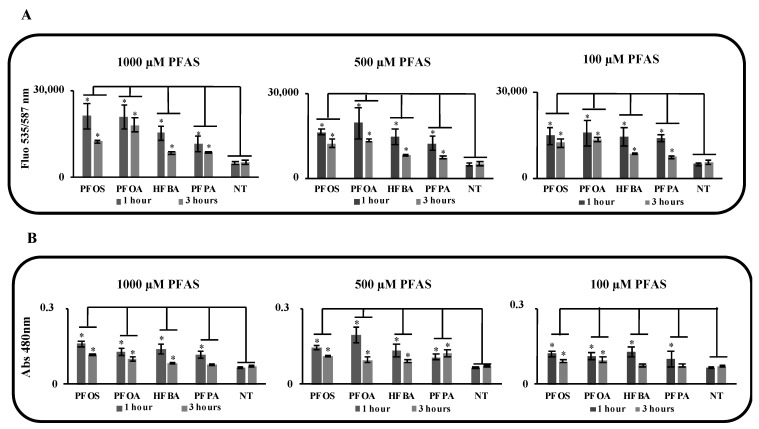
Evaluation of hepatotoxicity of PFAS by measuring the enzyme activity of ALT and GDH biochemical markers on mouse liver organoids treated for 1 and 3 h with 1 mM, 500 µM and 100 µM. (**A**) ALT activity increased starting from the lowest concentration (100 µM) for both long and short PFASs. (**B**) GDH activity followed the same trend showing an increase from the lowest concentration of treatment. *p*-value was calculated vs. (NT) untreated samples (* indicates *p*-value < 0.01).

Exposure to pollutants may increase the activity of liver enzymes that are markers of hepatotoxicity. In order to define the health state of liver, the activity of these two key enzymes were evaluated at different time points and concentrations (Figure 3). ALT activity increased starting from the lowest concentration (100 µM) for both long- and short-chain PFASs (Figure 3A). GDH activity followed the same trend showing an increase from the lowest concentration of treatment (Figure 3B). The secretion of ALT and GDH was higher after one hour for all the doses and all the compounds, except for the production of GDH by PFPA that is the similar for 1 and 3 h.

**Figure 4:**
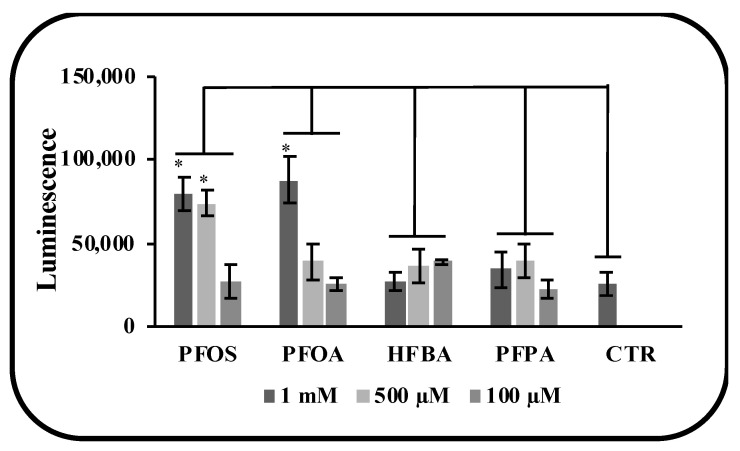
Activation of Caspase 3/7 as markers of apoptosis after 3 h of treatment with PFAS. Mouse liver organoids were treated for 3 with 1 mM, 500 µM and 100 µM. Results showed that only long chain PFAS induced apoptosis on organoids. *p*-value was calculated vs. (NT) untreated samples (* indicates *p*-value < 0.05).

In order to assess the levels of hepatotoxicity, the activation of apoptosis was studied (Figure 4). Caspase 3 and 7, two key proteins of the apoptotic program, were analyzed at different concentrations of PFASs. Only long chain PFASs activated the apoptosis but at very high concentration (1000 µM). Short chain PFASs did not show a trend of activation in the range of 100–1000 µM.

## 4. Discussion

The adverse effects of long-chain PFAS is well recognized and attested by numerous studies. Exposure to PFOA and PFOS is associated to different health problems including developmental effects [36], immunotoxicity [37], cardiotoxicity [38], pancreatic [39,40,41] and liver [42,43] damage, both in human and in animal models. Since PFASs accumulate in blood serum, liver, heart and kidney, these organs are constantly in contact with the toxicants and under stress conditions [44,45]. The acute toxicity tests performed on mouse liver organoids for both long- (PFOA and PFOS) and short-chain (HFBA and PFPA) PFAS displayed negative effects, with a marked disruption associated to long-chain compounds. The results obtained for PFOA and PFOS organoids are compatible with the results recorded on rats by Cui et al. [45]. The latter recorded accumulation of PFOA and PFOS with concentrations in liver of 218–196 and 345–648 ppm, respectively, on rats exposed to 5–20 (mg Kg^−1^d^−1^) for 28 days. The liver concentrations recorded by Cui et al. [45] were within the range implemented in this study of 500–1000 µM for PFOA and PFOS (equivalent to 207–414 ppm and 269–538 ppm, respectively) and in this range liver tumefaction and discoloration were recorded associated with major histological alterations. Rats treated with high doses of PFOA presented severe hepatotoxic symptoms including hepatocellular hypertrophy, hepatomegaly, microvesicular presence and necrosis [46,47]. Histological observations highlighted the hepatocytes with prominent smooth endoplasmic reticulum, hyper-eosinophilia and a granular or “ground glass” appearance within the cytoplasm indicative of enzyme reduction. Accordingly, the key liver enzymes ALT and GDH displayed in the organoids compromised functionality starting from the lowest tested concentration of 100 µM both from PFOS (53 ppm) and PFOA (41 ppm).

Toxicological data in rats demonstrated that long-term exposure to PFOA induced the formation of liver adenoma, Leydig cell adenomas, and pancreatic acinar cell tumors, the so called “tumor triad” [46,48,49]. A similar result was obtained with the use of PFOS regarding the significant increase in hepatocellular adenoma [47]. PFOA induces hepatocellular adenomas and hepatocyte hypertrophy, bile duct hyperplasia, and hematopoietic cell proliferation in mice after prenatal exposure through PPARα-independent pathways [50]. Transcriptomic analysis of mice treated with PFOS during embryonic development showed alteration of signaling pathways involved in liver cancer including Wnt/ßcatenin and CD44 adhesion molecule, a marker for stem cells [42].

Regarding the role of short-chain PFAS at the cellular level, toxicological studies carried out on rats for HFBA ammonium salts in 28 days and 90 days treatment periods [47] were reported to be generally mild and reversible upon cessation of the treatment. Current results attested to a lower toxicity on mouse liver organoids for the two short-chain compounds tested (HFBA, PFPA) in comparison with the long chain counterparts, nonetheless aberrant cytological features and enzyme disruption were recorded, especially by HFBA. This seems to be in accordance with the statements of Liu et al. [51] that reported cytotoxicity on human stem cells as well as potential developmental adipogenic and osteogenic toxicities of four short-chain PFASs at higher doses in comparison with PFOS and PFOA in the range of 0–300 µM. The tests performed by Croce et al. [52] on rat thyroid FRTL-5 cells reported as none of the tested short-chain PFAS produced any cytotoxic effect at concentrations up to 100 μM as found on mouse liver organoids although the monitoring of enzymatic activity on the organoids allowed to find alterations already from 100 µM acting as an early warning signal of disfunction. In the paper of Sheng et al. [53], the cytotoxicity of several fluorinated short-chain alternatives was tested on a human liver cell line HL-7702 and compared with PFOA and PFOS assessing a weaker cytotoxicity of the short chain compounds. Moreover, the authors highlighted as the backbone lengths of the PFAS influence their interaction and binding with proteins: with longer backbone lengths, a greater change in protein secondary structure was reported during binding and for chemicals with the same backbone length, atom types and branches determined the changes in protein structure [53]. About this point, Stefani et al. [54] by exposure experiments on flies, also hypothesized that the functional group (i.e., sulfonate) is expected as a more effective factor in inducing genotoxicity than the fluorinated chain length, it is therefore reasonable to expect not only a lower toxicity in general but also a lower genotoxicity of the short chains investigated here compared to sulfonated counterparts. Additionally higher toxicity of sulphonated PFAS was reported by Ulhaq et al. [55] on zebrafish. The conformational alterations in proteins by PFAS binding can severely compromise the enzyme functionality, additionally the toxicity of PFAS was reported to be correlated with carbon chain length by Mahapatra et al. [56] on a zebrafish liver cells. Following this conceptual thread, the short-chain PFAS when bound to enzymes could alter their structure less than long-chain PFAS because of their smaller steric hindrance inducing a milder loss of functionality and consequently producing a lower toxic effect. The lower toxicity for the tested short-chain compounds is expected to be accompanied by a lower permanence of HFBA and PFPA in the human body in comparison with PFOS and PFOA. A general pattern of increasing half-lives in humans was reported with increasing chain length with a respective half-life for PFOS and PFOA of 1.8–5.4 y and 2.3–3.8 years and no more than 62–70 days for HFBA [57,58,59,60]. Some authors highlighted critical considerations on the risk associated with short chain PFAS because the concentrations measured in blood and tissues both in animals and humans are many orders of magnitude lower (in the ppt/ppb range) than those expected to produce toxic effects (in the ppm range) [61,62,63,64]. The concentrations tested in this study for which effects occurred were also many times higher than those verified to be present in the environment and in humans. All of these factors contribute making HFBA and PFPA a safer alternative to long-chains such as PFOS and PFOA. Moreover, it is important to stress that considerable differences had been recorded between mouse and human, both in regard of the half-life of PFAS (such as PFOA maximum in humans; IARC, 2017) and in regard of the genotoxicity mechanisms [65]. The mechanisms of cell transformation were observed to be different in rodents and humans. Most of the studies highlighted how liver tumorigenesis in rodents derived from the non-genotoxic modes mediated by nuclear receptors, including PPARα, CAR, PXR, and the aryl hydrocarbon receptor (AhR), and especially PPARα was reported to be unlikely relevant in human cells [66]. Therefore, the results obtained for the mice must be cautiously considered in terms of human relevance, especially in genotoxicity and bioaccumulation studies. The application of organoid in this field is at the beginning and to confirm the oncogenetic properties of short-chain PFAS more studies are needed on many more compounds and mixtures on in vivo models. Moreover, some limitations should be addressed on organoids use such as the impossibility to carry out tests for chronic low-dose effects especially on short chain PFAS. On the other hand, if data will be confirmed, organoids could be used as a fast and reproducible platform to test acute toxicity of many PFAS and mixtures, whose effect is still largely unknown [26].

## 5. Conclusions

Several lines of evidence converge in describing a lower toxicity of two short-chain non sulfonated PFAS (HFBA and PFPA) in comparison with the sulfonated and long chain counterparts (PFOS and PFOA) on mouse liver organoids although some evidence of alteration occurred for the two investigated short-chain compounds. It was not possible to determine by EC50 an acute toxicity for HFBA and PFPA, however cytomorphological changes were appreciable at the lowest concentrations, although much less pronounced than for PFOS and PFOA and the enzymatic activity of key liver enzymes such as ALT and GDH displayed disruption from the lowest concentrations both by short and long chain PFAS. Eventually, apoptosis activation was recorded for PFOS and PFOA but not HFBA and PFPA also at the highest concentrations.

## Figures and Tables

**Table 1:** EC50 of PFAS on mouse liver organoids. Mouse liver organoids were treated with scalar (1:10) doses of PFASs starting from 1 mM, six serial dilutions for 96 h. NC (not calculable).

Compound	EC50 (µM)
PFOS (C_8_HF_17_O_3_S)	670 ± 30
PFOA (C_8_HF_15_O_2_)	895 ± 7
PFPA (CF_3_CF_2_CO)_2_O	NC
HFBA (C_4_HF_7_O_2_)	NC

## Data Availability

All data were published in the paper.
